# Serum concentrations of active tamoxifen metabolites predict long-term survival in adjuvantly treated breast cancer patients

**DOI:** 10.1186/s13058-017-0916-4

**Published:** 2017-11-28

**Authors:** Thomas Helland, Nina Henne, Ersilia Bifulco, Bjørn Naume, Elin Borgen, Vessela N. Kristensen, Jan T. Kvaløy, Timothy L. Lash, Grethe I. G. Alnæs, Ron H. van Schaik, Emiel A. M. Janssen, Steinar Hustad, Ernst A. Lien, Gunnar Mellgren, Håvard Søiland

**Affiliations:** 1 Hormone Laboratory, Haukeland University Hospital, Bergen, Norway; 2 Department of Clinical Science, University of Bergen, Bergen, Norway; 3 Core Facility for Metabolomics, University of Bergen, Bergen, Norway; 4 Department of Oncology, Division of Cancer Medicine, Oslo University Hospital, Oslo, Norway; 5 Institute of Clinical Medicine, Faculty of Medicine, University of Oslo, Oslo, Norway; 6 Pathology Department, Radium Hospital, Oslo University Hospital, Oslo, Norway; 7 Department of Cancer Genetics, Institute for Cancer Research, Oslo University Hospital Radiumhospitalet, Oslo, Norway; 8 Department of Mathematics and Natural Science, University of Stavanger, Stavanger, Norway; 9 Department of Research, Stavanger University Hospital, Stavanger, Norway; 10 Department of Epidemiology, Rollins School of Public Health, Winship Cancer Institute, Emory University, Atlanta, USA; 11 Expert Center Pharmacogenetics, Department of Clinical Chemistry, Erasmus University Medical Center, Rotterdam, The Netherlands; 12 Department of Pathology, Stavanger University Hospital, Stavanger, Norway; 13 Department of Surgery, Section of Breast and Endocrine Surgery, Stavanger University Hospital, Stavanger, Norway

**Keywords:** Tamoxifen, Adjuvant, Metabolism, Survival, CYP2D6, Endoxifen, 4OHtam, Breast cancer, Prognosis

## Abstract

**Background:**

Controversies exist as to whether the genetic polymorphisms of the enzymes responsible for the metabolism of tamoxifen can predict breast cancer outcome in patients using adjuvant tamoxifen. Direct measurement of concentrations of active tamoxifen metabolites in serum may be a more biological plausible and robust approach. We have investigated the association between *CYP2D6* genotypes, serum concentrations of active tamoxifen metabolites, and long-term outcome in tamoxifen treated breast cancer patients.

**Methods:**

From an original observational study comprising 817 breast cancer patients, 99 women with operable breast cancer were retrospectively included in the present study. This cohort of patients were adjuvantly treated with tamoxifen, had provided serum samples suitable for measuring tamoxifen metabolites, and were relapse-free at 3 years after the primary treatment commenced. The median follow-up time from this entry point to breast cancer death was 13.9 years. Patients were *CYP2D6* genotyped and grouped into four CYP2D6 phenotype groups (Ultra rapid, extensive, intermediate, and poor metabolizers). Tamoxifen and nine metabolites were quantified in serum (*n* = 86) and compared with CYP2D6 phenotype groups and outcome.

**Results:**

Breast cancer patients with low concentrations of Z-4-hydroxy-tamoxifen (Z-4OHtam; ≤ 3.26 nM) had a breast cancer-specific survival (BCSS) of 60% compared to 84% in patients with Z-4OHtam concentrations > 3.26 nM (*p* = 0.020, log-rank hazard ratio (HR) = 3.56, 95% confidence interval (CI) = 1.14–11.07). For patients with Z-4-hydroxy-N-desmethyl-tamoxifen (Z-endoxifen) levels ≤ 9.00 nM BCSS was 57% compared to 84% for patients with concentrations > 9.00 nM (*p* = 0.029, HR = 3.73, 95% CI = 1.05–13.22). Low concentrations of Z-4OHtam and Z-endoxifen were associated with poorer survival also after adjusting for clinically relevant variables (HR = 4.27, 95% CI = 1.35–13.58, and HR = 3.70, 95% CI = 1.03–13.25, respectively). Overall survival analysis showed similar survival differences for both active metabolites. The Antiestrogen Activity Score showed comparable effects, but did not improve the prognostic information.

**Conclusions:**

Patients with Z-4OHtam and Z-endoxifen concentrations lower than 3.26 nM or 9.00 nM, respectively, showed an adverse outcome. Our results suggest that direct measurement of active tamoxifen metabolite concentrations could be of clinical value. Validation in larger study cohorts is warranted.

**Electronic supplementary material:**

The online version of this article (doi:10.1186/s13058-017-0916-4) contains supplementary material, which is available to authorized users.

## Background

Tamoxifen is a selective estrogen receptor modulator used for adjuvant treatment of luminal (estrogen receptor (ER)-positive and/or progesterone receptor (PR)-positive) breast cancer (BC) subtypes. Tamoxifen is the oldest and most prescribed endocrine BC drug and has been shown to reduce BC mortality by 31% [1] and BC recurrence by 50% [2]. Tamoxifen is a widely used endocrine adjuvant treatment option among pre-menopausal BC patients, with therapy durations of up to 10 years [3, 4]. Post-menopausal BC patients are mainly given aromatase inhibitors (AIs) for 5 years, in combination with tamoxifen for a 3–5 year period, or tamoxifen monotherapy for 10 years if the side effects from AIs are too bothersome [5]. Hence, tamoxifen is still an important drug in the management of BC. However, interpatient variability in the anti-ER response and adverse effects are common. Within 15 years of primary surgery one-third of BC patients receiving tamoxifen will have relapsed [1].

The interpatient variability in the clinical response to tamoxifen has been suggested to be connected to its enzymatic conversion into active metabolites. Several of these activating enzymes are polymorphic, including cytochrome P450 2D6 (CYP2D6), as combinations of the *CYP2D6* alleles have been related to various kinetic activity levels of the enzyme. CYP2D6 is a key enzyme in the formation of the two active metabolites, Z-4-hydroxy-N-desmethyl-tamoxifen (Z-4OHNDtam, also known as Z-endoxifen) and Z-4-hydroxy-tamoxifen (Z-4OHtam) [6], and concentrations of these two active metabolites have been found to be associated with *CYP2D6* genotypes [7, 8]. Z-endoxifen and Z-4OHtam are 30- to100-fold more potent anti-ER inhibitors than the mother drug tamoxifen [9]. Endoxifen is present at up to 10 times higher plasma concentrations than 4OHtam and is therefore regarded as the most powerful metabolite [6]. After Goetz et al. in 2005 reported an association between the CYP2D6 poor metabolizer (PM) phenotype and higher risk of relapse among tamoxifen users [10], several reports have been published on *CYP2D6* genotype and outcome. However, the various studies have reported contradictory results and more knowledge is required in order to make any conclusions [11–14].

An alternative approach would be to measure the concentrations of the active metabolites directly in serum and associate them with breast cancer outcomes. As the active metabolites are strong ER ligands, their serum levels may better reflect the functional anti-estrogenic effects in patients treated with tamoxifen. Recently, methods to separate the Z-isomers (Z-endoxifen and Z-4OHtam) from the less active or inactive isomers have been developed [15]. The additive anti-ER effect from tamoxifen metabolites and isomers with various affinity to the ER may also be of importance to estimate the resultant effect of tamoxifen itself and all active tamoxifen metabolites [16].

In the present study, we have determined the *CYP2D6* genotypes and serum concentrations of tamoxifen and nine metabolites in 99 BC patients with a long-term follow-up. Our aim was to investigate the predictive value of direct measurements of active serum tamoxifen metabolites in patients with operable breast cancers and to compare these results with the *CYP2D6* genotyping method. We hypothesized that the genotype approach is inferior to direct measurement of tamoxifen metabolites regarding prediction of prognosis, and that patients with low serum levels of active tamoxifen metabolites will have poorer prognosis.

## Methods

In this retrospective observational study the primary objective was to compare the prognostic value of direct measurements of tamoxifen metabolites in serum with *CYP2D6* genotyping in 99 operable breast cancer patients. The secondary objective was to investigate the associations between concentrations of active tamoxifen metabolites and CYP2D6 phenotypes.

### Patients

Between May 1995 and December 1998, 817 patients were studied in a population-based observational micro-metastasis study [17] in Oslo, Norway. The patients were treated according to the national guidelines at the time. All patients with hormone receptor-positive tumors received 20 mg tamoxifen daily for 5 years. The tumor was defined as hormone receptor positive if ≥ 10% of the cells were positive for ER or PR by immunohistochemistry analysis.

From this original study population, serum was drawn from 356 relapse-free patients 3 years after inclusion. Of these, 99 operable BC patients comprising T1/T2 tumors were adjuvantly treated with tamoxifen and included in the present study. The demographic and clinical characteristics are presented in Table 1. The median follow-up time for breast cancer death from this entry time was 13.9 years (range 0.6–16.5 years). The present study population of 99 patients did not differ from the relapse-free cohort [17] with regard to clinical and tumor biological variables other than the treatment selection (Table 1).

**Table 1:** Patient demographics and characteristics

Characteristics	Present study population (*n* = 99)	Relapse free at 3 years (*n* = 356)	Differences between the groups (*P* values)
Age at diagnosis (years)			
Mean (median)	58 (56)	57 (56)	
Range	34–78	28–85	0.380
Menopause status, *n* (%)			
Pre (< 55 years)	40 (40%)	151 (42%)	
Post (≥ 55 years)	59 (60%)	205 (58%)	0.710
Histology, *n* (%)			
IDC	74 (75%)	251 (76%)	0.109
ILC	24 (24%)	69 (19%)	
Other infiltrating cancer	1 (1%)	16 (5%)	
Tumor size, *n* (%)			
pT1	50 (51%)	253 (71%)	< 0.001*
pT2	49 (49%)	87 (25%)	
pT3	–	12 (3%)	
pT4	–	0 (0%)	
pTx	–	3 (1%)	
Tumor grade, *n* (%)			
G1	18 (18%)	110 (31%)	0.009
G2	67 (68%)	184 (52%)	
G3	12 (12%)	56 (15%)	
Not reported	2 (2%)	6 (2%)	
Node status, *n* (%)			
Positive	57 (58%)	93 (71%)	< 0.001
Negative	41 (41%)	257 (27%)	
Not reported	1 (1%)	6 (2%)	
HER2/neu status, *n* (%)			
HER2^+^	7 (7%)	36 (10%)	0.193
HER2^–^	89 (90%)	298 (84%)	
Unknown	3 (3%)	22 (6%)	

Comparison of the demographic and clinical characteristics between the patients in the present study population and 356 relapse-free patients from the original population [17]

The present study population comprises more patients with pT2 tumors, higher grade, and node-positive status due to treatment selection

*The present study population only included operable breast cancer patients; therefore *p* value of tumor size comparison is between pT1 and pT2 populations

*IDC* invasive ductal carcinoma, *ILC* invasive lobular carcinoma, *pT* pathological tumor size

### *CYP2D6* genotyping and classification of CYP2D6 phenotype groups

DNA was isolated from the blood or bone marrow using the Gentra Puregene Blood kit (Qiagen, Hilden, Germany) or an automated phenol-chloroform procedure. The *CYP2D6* genotype determination was performed at the Expert Center for Pharmacogenetics, Department of Clinical Chemistry, Erasmus University Medical Center, Rotterdam, The Netherlands, using the CE-IVD approved INFINITI® CYP450 2D6I Assay (Autogenomics, Carlsbad, CA, USA) and verified using the Taqman DME assay (Thermo Fisher Scientific, Waltham, MA, USA) according to validated standard operating procedures in an ISO15189-certified laboratory. INFINITI detects 15 variant alleles (Additional file 1: Table S1) and the *CYP2D6* genotypes of the patients were determined based on the combination of wild-type (wt) and variant-type (vt) alleles and translated into four predicted CYP2D6 phenotype groups: ultra-rapid metabolizers (UM; gene duplication positive, no inactive variants), extensive/normal metabolizers (EM; no variants or only one decreased activity allele), intermediate metabolizers (IM; two decreased activity alleles or one active and one inactive allele), and poor metabolizers (PM; two inactive alleles).

### Determination of tamoxifen metabolites by liquid chromatography-tandem mass spectrometry (LC-MS/MS)

We developed a LC-MS/MS method to quantify tamoxifen and nine of its metabolites in human serum. All metabolites and four de uterated internal standards were obtained commercially (Additional file 2: Table S2). Calibrators were created from pooled human serum of three male and six female non-tamoxifen users to which tamoxifen metabolites were added at seven concentrations (Additional file 3: Table S3).

Serum samples (50 μl) containing tamoxifen metabolites were processed using a Hamilton STAR pipetting robot (Bonaduz, Switzerland). Serum proteins were precipitated by adding 500 μL acetonitrile containing internal standards to the samples; 350 μL of the supernatant was evaporated to dryness under a nitrogen flow and subsequently reconstituted in 500 μL methanol and diluted 1 to 25 in water:methanol (20:80, v:v) before being subjected to LC-MS/MS analysis.

An Aquity UPLC system from Waters (MA, USA) with a thermostated column oven set at 50 °C was used to chromatographically separate the compounds; 25 μL of sample was injected onto a 100-mm BEH Phenyl column with a 2.1 mm internal diameter and 1.7 μm particle size (Waters, Milford, MA, USA). The column was developed by a weak mobile phase (A) consisting of water, and a strong mobile phase (B) consisting of methanol, both buffered with 0.01% formic acid. All gradient steps were linear, and the flow rate was 300 μL/min. The following gradient was used: 0–0.5 min: 95% A and 5% B; 1 min: 65% A and 35% B; 4 min: 10% A and 90% B; 4.5–8 min: 100% B; 8.1–9 min: 90% A and 10% B.

The LC system was coupled to a Xevo TQ-S tandem mass spectrometer (Waters, Milford, MA, USA) equipped with an atmospheric pressure photoionization source (APPI). All compounds were analyzed in the positive mode. Additional file 4: Table S4 shows retention times and compound-dependent settings for the tamoxifen metabolites.

### Validation of the LC-MS/MS method

**Fig. 1:**
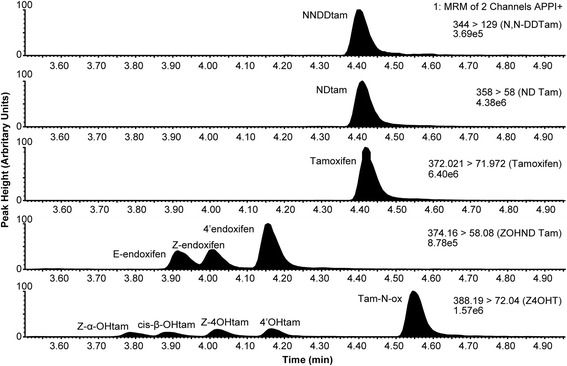
MRM transitions of tamoxifen and its metabolites. The chromatograms are obtained by analyzing the second point of the calibration curve. The chromatographic separation of isomers of 4OHtam and endoxifen are shown in the lowest and second lowest panels, respectively. *APPI* atmospheric pressure photoionization, *MRM* multiple reaction monitoring

The selectivity of the method is demonstrated in Fig. 1 as it separated the active Z-isomers of 4OHtam and endoxifen from its less active Z’-isomers and inactive E-isomers. Total analytical run-time was 9 min and the sample volume of serum was 50 μL. Accuracy and imprecision was well within the acceptance criteria defined by regulatory guidelines (Food and Drug Administration (FDA), Rockville, MD, USA; 2002). The method was linear for all analytes (Additional file 5: Table S5). For medium concentrations, imprecision (intra- and inter-day CV %) was within 9% and accuracies were in the range 95–106% (Additional file 3: Table S3) for all metabolites except cis-β-OHtam and z-α-OHtam, which had imprecision within 15% accuracies in the range 87–109%. Cis-β-OHtam and z-α-OHtam were not detected in patient samples.

### Data analyses and statistics

SPSS statistical software, version 23 (SPSS, Inc., Chicago, IL, USA), and MedCalc for Windows, version 16.4.3 (MedCalc Software, Ostend, Belgium), were used for the basic statistical calculations.

Supervised cut-off values for Z-endoxifen (9.00 nM), Z-4OHtam (3.26 nM), and Antiestrogenic Activity Score (AAS) (16.7) were identified by multivariable Cox approach as described in Additional file 6.

The present study included patients that had survived the first three 3 years after surgery without experiencing any relapse. The analysis is thus conditional on 3 year relapse-free survival, and in the survival analysis 3 years after surgery is used as the time origin to partially address the pitfall of immortal person time bias [18–20]. Breast cancer-specific survival (BCSS) was defined as the time from the primary surgery until death from breast cancer. Cause of death was provided from the hospital records, and in a few cases also by information from the patient's general physician.

Survival estimates were calculated by the Kaplan-Meier method. Univariable tests for survival differences in categorical variables were performed by the log-rank test or the log-rank test for trend as appropriate. Multivariable regression analysis for clinically relevant variables was performed using the Cox proportional hazards method. Chi-square, Mann-Whitney *U* test or Kruskal-Wallis were used for comparisons between groups as needed. Fisher’s exact test was used when appropriate. Two-tailed *P* values < 0.05 were considered statistically significant.

To estimate the resultant ER blockade of tamoxifen itself and the various active tamoxifen metabolites we used the AAS as previously described [16]. In short, the estimation of the AAS was based on the serum concentrations of the various active tamoxifen metabolites and their relative affinity to the ER by the following algorithm: 0.01 × [Tamoxifen] + 1 × [Z-endoxifen + Z-4OHtam] + 0.1 × [Z′-endoxifen + Z′-4OHtam].

## Results

### *CYP2D6* genotyping and quantification of tamoxifen metabolites in serum

*CYP2D6* allele frequencies are shown in Additional file 7: Table S6 and the most frequent genetic variants *1, *2, *4 and *41 were in Hardy-Weinberg equilibrium (HWE). The frequency of the remaining 5 alleles (*3, *5, *6, *9, and *10) were too rare in our study cohort to perform a HWE calculation. Ninety-one patients were successfully *CYP2D6* genotyped and the phenotypes were distributed as follows: 4 (4.4%) ultra-rapid metabolizers, 43 (47.3%) extensive/normal metabolizers, 36 (39.6%) intermediate metabolizers, and 8 (8.8%) poor metabolizers. Eight patients were excluded from *CYPD6* analysis due to inadequate volumes of blood/bone marrow for DNA extraction or because of poor quality of DNA.

Concentrations of tamoxifen and nine metabolites were measured using LC-MS/MS. All patient serum samples were run in duplicate (Table 2). The mean and median concentrations of tamoxifen and the nine metabolites for the 86 patients are shown in Table 2. Cis-β-OHtam and z-α-OHtam were included for separation of the hydroxylated metabolites [21] and were not detectable in patient samples. As shown before [22], large inter-individual variations in the concentrations of tamoxifen metabolites were observed between patients (Table 2). Thirteen patients had concentrations below the lower limits of quantification (LLQ) for tamoxifen and all the nine metabolites. These patients were regarded as non-adherent and excluded from the present study, leaving 86 patients for further analyses.

**Table 2:** Concentrations of tamoxifen and nine metabolites in 86 breast cancer patients

Analyte	Mean (median) serum concentration (nM)	Analytical CV %	Interpatient variability CV %
Tamoxifen	322.2 (287.5)	6.61	45.46
NDtam	723.2 (689.0)	9.55	44.75
Z-4OHNDtam	30.11 (28.15)	6.46	59.01
4'OHNDtam	30.08 (28.13)	8.18	38.09
Z-4OHtam	5.67 (5.30)	6.03	42.32
4'OHtam	7.64 (7.20)	7.54	39.34
Tam-N-ox	119.6 (97.52)	11.04	60.12
NNDDtam	92.69 (81.17)	11.14	50.80
cis-β-OHtam	ND	–	–
z-α-OHtam	ND	–	–

Samples were run in duplicate

Thirteen patients with metabolite levels below the limit of detection are not included in the calculations, leaving 86 patients for further analysis

Analytical CV % indicates average CV between two replicate samples for all patients

*CV* coefficient of variation, *ND* not detected

### Associations between CYP2D6 phenotype groups and concentrations of tamoxifen metabolites

**Fig. 2:**
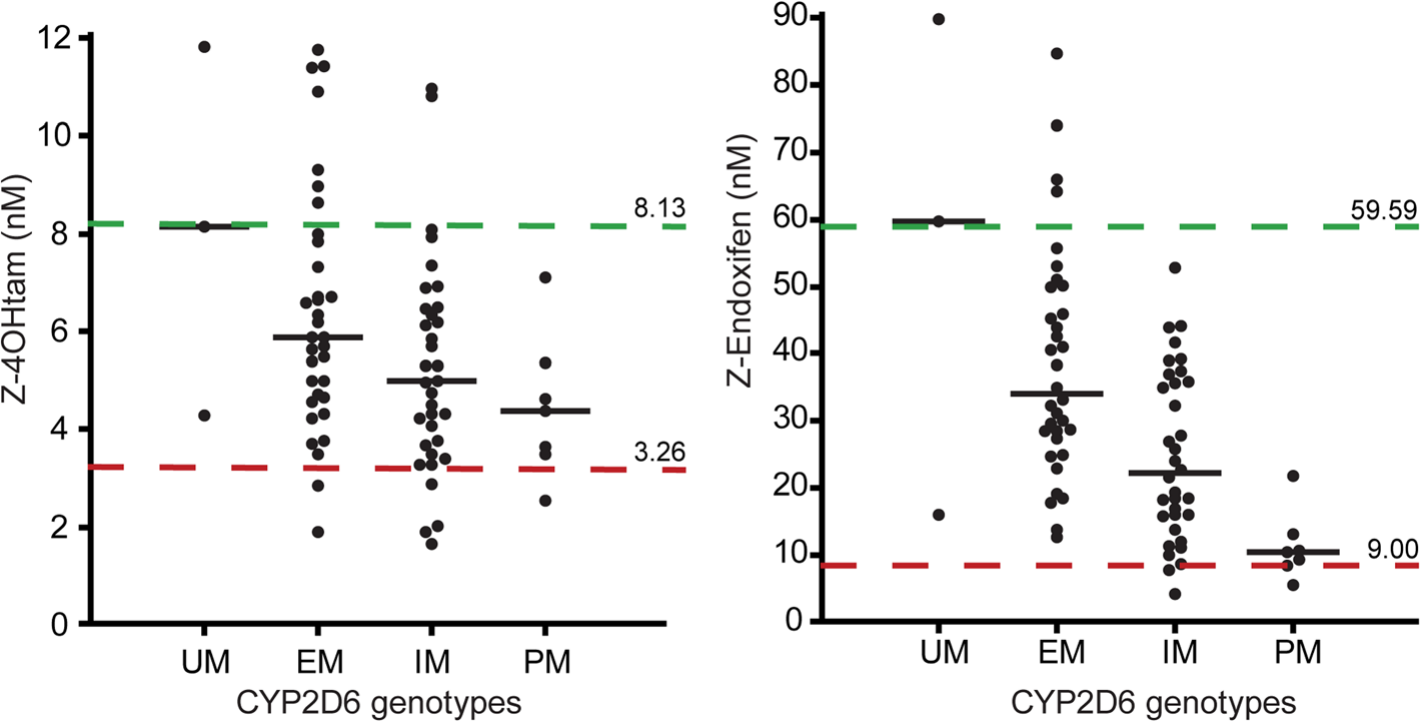
Z4OHtam and Z-endoxifen concentrations compared by CYP2D6 phenotype groups. Impaired *CYP2D6* function correlates with lower levels of Z-4OHtam and Z-endoxifen (*p* = 0.05 and *p* <0.001, respectively; Kruskal-Wallis). Cut-off values representing patients with high levels (*green line*) and low levels (*red line*) of active metabolites are shown. *EM* extensive metabolizer, *IM* intermediate metabolizer, *PM* poor metabolizer, *UM* ultra-rapid metabolizer

The median values for all metabolite concentrations stratified by CYP2D6 phenotype groups are shown in Additional file 8: Table S7. An association between declining concentration levels and decreased CYP2D6 function was observed for Z-4OHtam and Z-endoxifen (*p* = 0.05 and *p* < 0.001, respectively; Kruskal-Wallis) (Fig. 2). Notably, there is a wide spread of levels of active metabolites within each CYP2D6 phenotype group and also a considerable overlap between them; for example, use of the 3.26 nM (red line) and 8.13 nM (green line) cut-off values for Z-4OHtam will include patients from three CYP2D6 phenotype groups (Fig. 2). None of the other metabolite concentrations showed an association with CYP2D6 phenotype.

### Breast cancer outcome in association with CYP2D6 phenotype and active tamoxifen metabolite concentrations

**Fig. 3:**
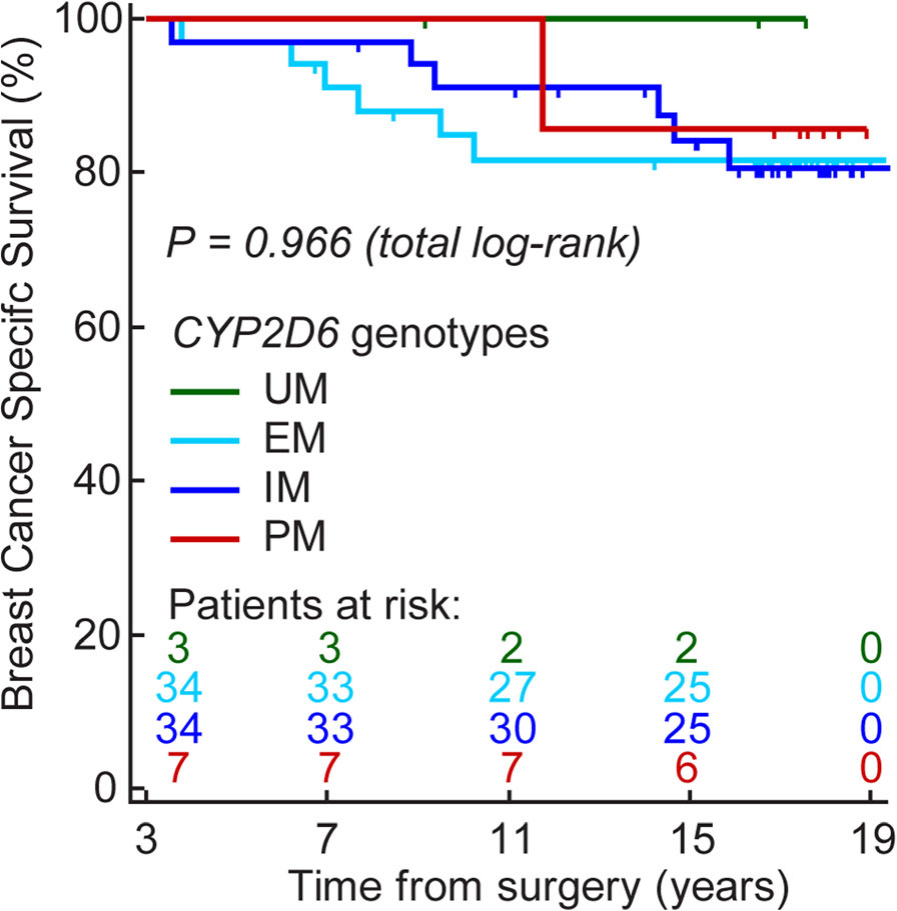
Kaplan-Meier plot of BCSS for CYP2D6 phenotypes. Patients are grouped according to CYP2D6 phenotype group as indicated by the colored lines. Time starting at 3 years after surgery. *EM* extensive metabolizer, *IM* intermediate metabolizer, *PM* poor metabolizer, *UM* ultra-rapid metabolizer

To investigate the association between CYP2D6 phenotype and survival, a Kaplan-Meier linear trend analysis comparing the survival of the four CYP2D6 phenotype groups (UM, EM, IM, and PM) was performed (Fig. 3). No significant association was observed (*p* = 0.966, total log-rank). However, using EM as a reference we achieved 80% power to detect hazard ratios (HRs) of 3.3, 10, and 35 for IM, PM, and UM, respectively. Therefore, the result should be interpreted with care. Notably, the excluded non-adherent patients (*n* = 13) were evenly distributed among the various CYP2D6 phenotype groups, and including them did not change the results.

**Fig. 4:**
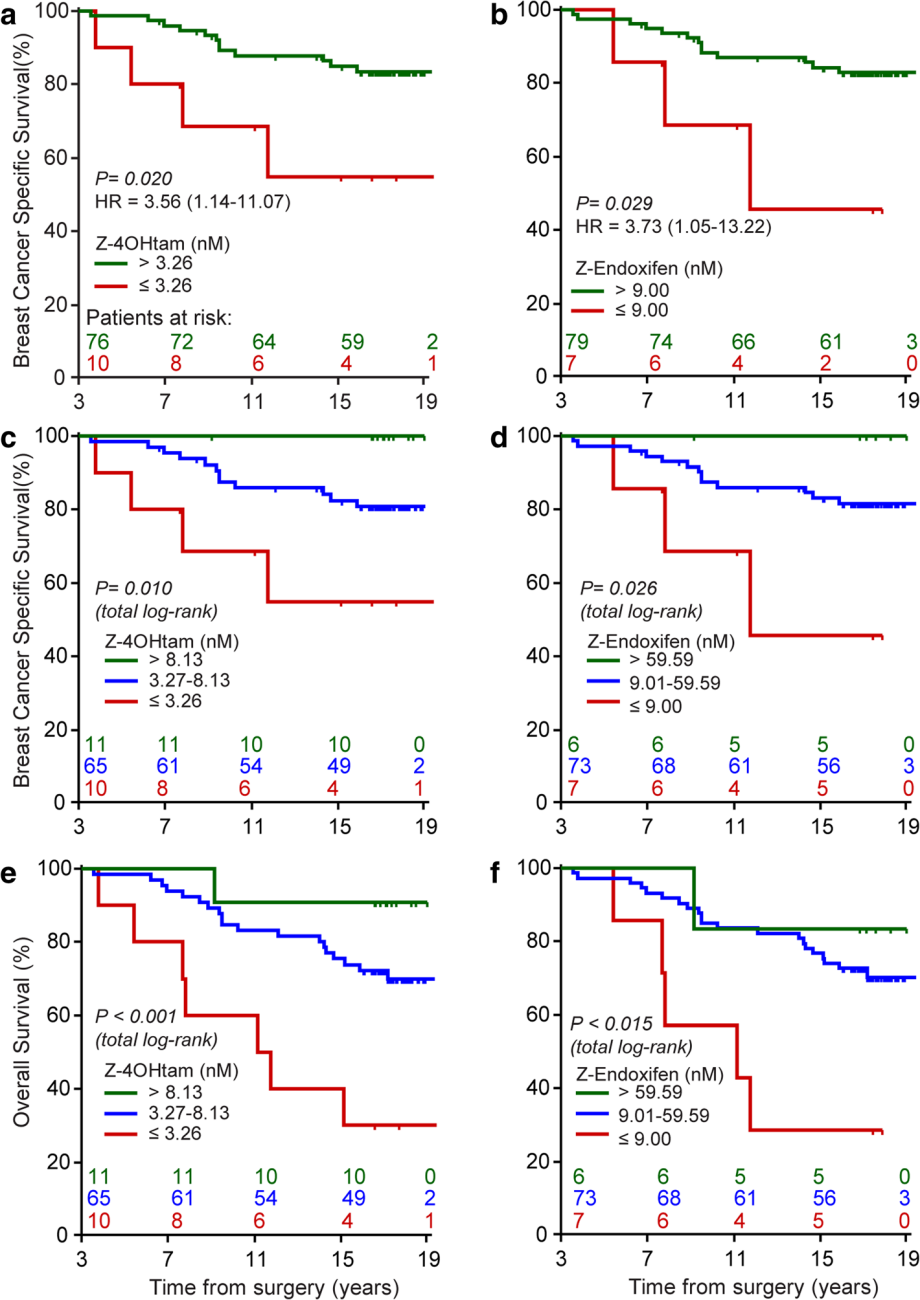
Kaplan-Meier plots of BCSS and overall survival for concentrations of active tamoxifen metabolites. Patients are grouped according to concentrations of active metabolites as indicated by colored lines. Time starting at 3 years after surgery. **a,b** BCSS for Z-4OHtam and Z-endoxifen at concentrations above and below 3.26 nM and 9.00 nM, respectively. **c,d** BCSS for Z-4OHtam and Z-endoxifen at three serum concentrations: low, intermediate, and high levels, as shown in the figure. **e,f** Overall survival for Z-4OHtam and Z-endoxifen at the same three concentrations as shown in **c** and **d**. *HR* hazard ratio

A Cox log-linear trend analysis controlling for age, tumor size, grade, node status, ER, PR, and chemotherapy was performed to investigate the association between concentrations of tamoxifen metabolites and outcome. We identified a log-linear relationship between Z-4OHtam and BCSS (*p* = 0.044, HR = 0.75, 95% confidence interval (CI) = 0.56–0.99), indicating a 0.25 reduction in hazard for each unit (1 nM) increase in Z-4OHtam. There was no log-linear association between Z-endoxifen or the remaining metabolites and breast cancer outcome. We further wanted to explore the possibility of an association between survival and concentration thresholds for the active metabolites Z-4OHtam and Z-endoxifen. We identified supervised cut-off values representing low concentrations for Z-4OHtam (3.26 nM) and Z-endoxifen (9.00 nM) as described in the Methods section and performed univariable survival analyses (Fig. 4a and b). For Z-4OHtam the BCSS was 60% vs. 84% for the ≤ 3.26 nM and > 3.26 nM groups, respectively (*p* = 0.020; log-rank HR = 3.56, 95% CI = 1.14–11.07). For Z-endoxifen we observed a BCSS of 57% vs. 84% for the ≤ 9.00 nM and > 9.00 nM groups, respectively (*p* = 0.029; log-rank HR = 3.73, 95% CI = 1.05–13.22). Adjustment for age, tumor size, nodal status, histological grade, ER and PR status, and chemotherapy given left Z-4OH tam and Z-endoxifen as the only factors in the final models with HR = 4.27 (95% CI = 1.35–13.58) and HR = 3.70 (95% CI = 1.03–13.25), respectively.

The Z’ isomers of the active metabolites also have anti-estrogenic activity and, since our LC-MS/MS was able to measure the Z and Z’ isomers of 4OHtam and endoxifen separately, we were able to calculate the AAS score (as described in Additional file 6). We further identified threshold values representing patients with low and high AAS and showed a BCSS of 57% for patients with AAS ≤ 16.7 compared to 84% in patients with ASS > 16.7 (*p* = 0.026, HR = 3.81, 95% CI = 1.07–13.56) (Additional file 9: Figure S1). Adjusting for the same variables as mentioned above, AAS was the only factor associated with BCSS (*p* = 0.041, HR = 3.80, 95% CI = 1.06–13.64). We also investigated the possible effect on outcome from tamoxifen itself, the two Z’-isomers alone, and the various non-active metabolites. No significant thresholds were identified.

In the analysis for overall survival (OS), Z-4OHtam, Z-endoxifen, and AAS were all significant in the univariable analysis (Table 3). When adjusting for the various clinico-pathological variables, tumor size, nodal status, and chemotherapy were added to the final models (Table 4).

**Table 3:** Univariable survival analyses of breast cancer-specific survival and overall survival

	Breast cancer-specific survival				Overall survival			
Factor	Event/at risk	HR	95% CI	*P*	Event/at risk	HR	95% CI	*P*
Tumor size								
pT1	7/44	1			11/44	1		
pT2	9/42	1.47	0.55–3.96	0.439	16/42	1.70	0.79–3.67	0.169
Node status								
pN0	4/33	1			9/33	1		
pN+	12/52	2.00	0.64 –6.19	0.222	18/52	1.33	0.60–2.96	0.484
Histological grade								
1	3/16	1			6/16	1		
2	10/57	0.94	0.26–3.42	0.924	16/57	0.76	0.30–1.94	0.564
3	3/11	1.48	0.30–7.35	0.631	5/11	1.30	0.40–4.28	0.661
Histological grade								
1 + 2	13/73	1			22/73	1		
3	3/11	1.56	0.44–5.46	0.335	5/11	1.61	0.61–4.25	0.335
Age								
< 55 years	7/35	1			7/35	1		
≥ 55 years	9/51	0.93	0.35–2.51	0.890	20/51	2.09	0.89–4.95	0.085
ER								
≥ 10%	15/75	1			26/75	1		
< 10%	1/7	0.63	0.08–4.73	0.646	1/7	0.36	0.05–2.65	0.294
PR								
≥ 10%	11/55	1			17/55	1		
< 10%	4/27	0.72	0.23–2.25	0.566	9/27	0.98	0.43–2.19	0.951
Chemotherapy*								
Yes	6/30	1			6/30	1		
No	10/56	0.93	0.34–2.57	0.893	21/56	1.97	0.80–4.89	0.135
AAS								
> 16.7	13/79	1			21/79	1		
≤ 16.7	3/7	3.81	1.07–13.56	0.026	6/7	5.37	2.14–13.51	< 0.001
Z-4OHtam								
> 3.26 nM	12/76	1			20/76	1		
≤ 3.26 nM	4/10	3.56	1.14–11.11	0.020	7/10	4.05	1.70–9.64	0.001
Z-endoxifen								
> 9.00 nM	13/79	1			22/79	1		
≤ 9.00 nM	3/7	3.73	1.05–13.23	0.029	5/7	4.03	1.51–10.74	0.003

* Did the patients receive chemotherapy according to the treatment guidelines at the time

*AAS* antiestrogenic activity score, *CI* confidence interval, *ER* estrogen receptor, *HR* hazard ratio, *pN* pathologic node status, *PR* progesterone receptor, *pT* pathologic tumor size

**Table 4:** Overall survival; multivariable analysis including Z-4OHtam, Z-endoxifen, and AAS

Continuous variables				Categorical variables				
Factor	HR per unit*	95% CI	*P*	Factor		HR	95% CI	*P*
Z-4OHtam				Z-4OHtam	> 3.26 nM	1		
Adjusted^†^	0.81	0.66–0.99	0.040		≤ 3.26 nM	4.86	1.88–12.54	0.001
Unadjusted	0.85	0.70–1.02	0.077	pT	1	1		
					2	2.59	1.11–6.05	0.028
				pN	Negative	1		
					Positive	2.89	1.12–7.49	0.029
				Chemotherapy	Yes	1		
					No	2.34	0.90– 6.11	0.083
Z-endoxifen				Z–endoxifen	> 9.00 nM	1		
Adjusted^†^	0.99	0.96–1.02	0.365		≤ 9.00 nM	5.65	2.00–16.00	0.001
Unadjusted	0.99	0.97–1.02	0.580	pT	1	1		
					2	2.44	1.08–5.49	0.032
				pN	Negative	1		
					Positive	2.40	0.99–5.80	0.052
				Chemotherapy	Yes	1		
					No	3.41	1.24–9.39	0.017
AAS				AAS	> 16.7	1		
Adjusted^†^	0.99	0.97–1.01	0.313		≤ 16.7	8.39	2.90–24.26	<0.001
Unadjusted	0.99	0.98–1.10	0.532	pT	1	1		
					2	2.60	1.15–5.91	0.022
				pN	Negative	1		
					Positive	3.40	1.26–9.15	0.016
				Chemotherapy	Yes	1		
					No	2.67	1.03–6.94	0.044

* Change in hazard ratio (HR) per 1 nM increase in serum concentration of Z-4OHtam, Z-endoxifen, change in HR per 1 unit Antiestrogenic Activity Score (AAS; dimensionless)

^†^ Adjusting variables: pT, pN, histological grade, estrogen receptor, progesterone receptor, age, and chemotherapy

*CI* confidence interval, *pN* pathologic node status, *pT* pathologic tumor size

The significant linear trend observed for Z-4OHtam encouraged us to also assess survival effects for very high levels of active metabolites. We therefore arbitrarily used the concentrations corresponding to the median concentrations for Z-4OHtam and Z-endoxifen ultra-rapid metabolizers (UM) as cut-off values, i.e., 8.13 nM and 59.59 nM, respectively (Fig. 2). Hence, patients were re-grouped into low, intermediate, and high serum concentrations of Z-4OHtam and Z-endoxifen, respectively (Fig. 4c and d). The Kaplan-Meier log-rank trend test demonstrated significant survival differences between these three subgroups for both metabolites (Z-4OHtam, *p* = 0.010; Z-endoxifen, *p* = 0.026) with no BCSS events for patients with high concentrations of active metabolites (Fig. 4c and d). The same differences were also observed in the overall survival analysis (Z-4OHtam, *p* = 0.002; Z-endoxifen, *p* = 0.014; log-rank trend) (Fig. 4e and f). Notably, the distribution of all the adjusted clinic-pathological variables were equal between the low and the high serum concentration subgroups.

## Discussion

In the present study we identified an association between CYP2D6 phenotype groups and the serum levels of active metabolites (Z-4OHtam and Z-endoxifen). However, we did not find an association between CYP2D6 phenotypes and breast cancer outcome (Fig. 3). The low power to detect a relevant survival difference between the CYP2D6 phenotype groups (i.e., HR between 1.5 and 2.5) is a possible explanation for its absent prognostic value in the present study. We further investigated the association between concentrations of active metabolites and breast cancer outcome, and this is to our knowledge the first study to report a relationship between low levels of the active tamoxifen metabolites and higher risk of *breast cancer death* (Fig. 4). The long follow-up time in our study allowed the use of breast cancer-specific survival as the clinical endpoint. We identified threshold values representing low and high levels of active metabolites. Notably, these cut-off values included patients from all CYP2D6 phenotype groups suggesting that the genotype approach results in grouping of patients with heterogeneous serum levels of active metabolites (Fig. 2).

To our knowledge, only three studies have analyzed the association between tamoxifen metabolite concentrations and *relapse of breast cancer* [23–25]. Madlensky et al. found a 30% higher risk of relapse in patients with low endoxifen levels (<16 nM) in patients grouped according to endoxifen quintiles [23]. In a recent study, a higher risk of distant relapse was observed in patients with low (<14.15 nM) vs high (>35 nM) Z-endoxifen levels when splitting the patients into endoxifen quartiles [24]. Both studies reported that the lowest quintile/quartile had the worst outcome, whereas the highest quintile/quartile had the best outcome. Thus, it seems that the use of active metabolite thresholds creates reproducible results in survival analyses probably due to grouping of patients that are homogeneous regarding the anti-ER effect. This is in line with our results since we also observed a favorable survival in breast cancer patients with high serum metabolite levels. Our high cut-off value is equal to the median concentrations of active metabolites in the UM group (Fig. 2), and other studies have shown that UM groups are often reported to be in the best prognostic range in the subgroup analyses [26]. In a third study, no association was found between endoxifen levels and breast cancer outcome in patients receiving low doses of tamoxifen (1 mg, 5 mg, and 10 mg) [25]. However, the authors speculate that sensitivity issues for detecting differences at very low concentrations may have clouded the results. In addition, preliminary results presented at ASCO 2016 [27] showed no association between endoxifen concentrations and BC outcome. However, this study included patients receiving 20 mg tamoxifen in the metastatic setting or as neoadjuvant treatment, a very different context often with developed endocrine resistance. Hence, this patient group is difficult to compare with the operable patients undergoing adjuvant tamoxifen treatment in our study. Interestingly, in a phase I study administering oral Z-endoxifen 160 mg daily in endocrine refractory metastatic breast cancer patients [28] the response rate on the tumor was 26% and the side effects were tolerable, with endoxifen concentrations up to 5200 nM maintained over 28 days. This study suggests that the concentrations of the active metabolites may be important for the apoptotic effect on breast cancer cells [28].

Here, we report for the first time an association between Z-4OHtam and BC outcome. Although endoxifen is present at higher serum concentrations than Z-4OHtam, their affinity to the ER is the same. Cross tabulation between Z-endoxifen (cut-off 9.0 nM) and Z-4OHtam (cut-off 3.26nM) shows that 50% of patients below the Z-4OHtam threshold were not identified by the Z-endoxifen threshold (Additional file 10: Table S8). This implies that measuring Z-4OHtam may be of clinical value. The Z’-isomers of the active metabolites also have a certain antiestrogenic effect. After calculation of tamoxifen and all active metabolites by means of the AAS score, we observed a significant association between low AAS score and worse BCSS (Additional file 9: Figure S1). However, using the AAS score was not superior to the use of Z-endoxifen and Z-4OHtam concentrations, strengthening previous observations that Z-endoxifen and Z-4OHtam are the most active tamoxifen metabolites. In line with our results, a recent study showed an aggregate effect of tamoxifen and three metabolites on breast cancer relapse [29] without providing additional prognostic information compared to the use of endoxifen levels alone.

Our supervised threshold for low concentrations of endoxifen identified in the present study (9.00 nM) is slightly lower compared to the un-supervised thresholds identified in previous studies (16 nM and 14.15 nM) [23, 24]. Using these cut-off values in the present study, we observed the same pattern with poorer survival for the lower concentration groups; however, significance was not reached. Thresholds will vary depending on the number of patients included in a study, the statistical methods to determine cut-off values [30], the underlying patient distribution [31], and the assay used to quantify the metabolites. Moreover, the threshold of a single metabolite in a clinical study (in-vivo setting) may be influenced by the relative concentrations of all the other metabolites present in the same environment. Thus, they will compete on the same binding site of the ER and contribute to the numeric difference in cut-off values.

Intriguingly, all the above three cut-off values identify a clinical relevant patient group with poor outcome in the lower concentration extremities of Z-endoxifen and Z-4OHtam. Admittedly, our supervised thresholds may also have inflated the *P* values [32] and exaggerated the survival differences between subgroups in the present study. Hence, the threshold values in this learning set must be interpreted with caution and validation of the thresholds in a larger independent material is warranted [33]. Importantly, consensus on the “correct clinical threshold” should aim to characterize patients with low benefit of tamoxifen with a certain safety margin to avoid under-treatment.

There are some limitations to the present study. First, our patient population of 86 patients is small. Despite the low number of patients, we were able to identify significant associations between Z-4OH tam and Z-endoxifen levels and outcome probably due to our long follow-up time (median 13.8 years). The low number of events in each subgroup calls for caution in interpreting the results and may explain the lack of statistical power to determine prognostic information from the CYP2D6 phenotype groups. Therefore, validation in larger study cohorts is warranted. Furthermore, information on long-term adherence (5 years) and co-medication, such as CYP2D6 inhibitors, would have strengthened our study. Entry of patients after a 3-year relapse-free period post-surgery has created loss of early endpoints occurring during the first 3 years of follow-up. This might have contributed to the observed loss of prognostic information of the proliferation-related variables such as pT, pN, and histological grade in these luminal breast cancer subtypes. This selection bias will favor patients with late events in the natural course of their disease. In patients with luminal breast cancers, approximately 75% of the breast cancer-related deaths occur after 3 years [34]. As the present study comprises only patients with this tumor type with a long-term follow-up (i.e., up to 16.5 years) we believe that our findings are of value for evaluating the 3-year conditional survival in this patient group.

## Conclusions

Although tamoxifen has been on the market for several decades and is the most used drug against breast cancer, its use may still be improved. The present study shows that tamoxifen metabolism may predict breast cancer outcome by measuring serum concentrations of active tamoxifen metabolites. Our results imply that patients with serum Z-endoxifen levels lower than 9.00 nM or Z-4OHtam levels lower than 3.26 nM have poorer long-term BCSS and OS compared to patient with levels above these thresholds. The results may translate into clinical practice by means of therapeutic drug monitoring, which represents a direct and applicable method to identify breast cancer patients with poor tamoxifen metabolism regardless of genotype and inhibiting drug interactions on the CYP enzymes [35]. Dose adjustment or a switch to an alternative endocrine treatment could avoid under-treatment of such patients [36]. Our findings need to be verified in larger studies, preferable in randomized trials with a long follow-up time.

## Additional files

Additional file 1: Table S1. Alleles analyzed by INFINITI®. (DOCX 14 kb)

Additional file 2: Table S2. Suppliers and catalog numbers for tamoxifen metabolites. (DOCX 14 kb)

Additional file 3: Table S3. Imprecision and accuracy. (DOCX 16 kb)

Additional file 4: Table S4. Retention times, molecular weights, and compound-dependent instrument settings. (DOCX 16 kb)

Additional file 5: Table S5. Linear dynamic range of the assay. (DOCX 14 kb)

Additional file 6: Additional methods. Description of methods to determine cut-off values. (DOCX 17 kb)

Additional file 7: Table S6. *CYP2D6* allele frequencies. (DOCX 13 kb)

Additional file 8: Table S7. Concentrations of tamoxifen metabolites stratified by metabolizer group. (DOCX 14 kb)

Additional file 9: Figure S1. Kaplan-Meier plot of breast cancer-specific survival according to Antiestrogenic Activity Score. (DOCX 109 kb)

Additional file 10: Table S8. Distribution of patients with high and low Z-endoxifen (cut-off 9.0 nM) among patients with high and low Z-4OHtam (cut-off 3.26 nM). (DOCX 13 kb)

## Abbreviations

AAS: Antiestrogenic Activity Score
AI: Aromatase inhibitor
BC: Breast cancer
BCSS: Breast cancer-specific survival
CI: Confidence interval
CYP2D6: Cytochrome P450 2D6
EM: Extensive/normal metabolizer
ER: Estrogen receptor
HR: Hazard ratio
HWE: Hardy-Weinberg equilibrium
IM: Intermediate metabolizer
LC-MS/MS: Liquid chromatography-tandem mass spectrometry
LLQ: Lower limits of quantification
OS: Overall survival
PM: Poor metabolizer
PR: Progesterone receptor
UM: Ultra-rapid metabolizer
vt: Variant type
wt: Wild-type
Z-4OHtam: Z-4-hydroxy-tamoxifen
Z-endoxifen: Z-4-hydroxy-N-desmethyl-tamoxifen
