# Supplementary material for: Serum concentrations of active tamoxifen metabolites predict long-term survival in adjuvantly treated breast cancer patients
Source: Breast Cancer Res. 2017 Nov 28;19:125. doi: 10.1186/s13058-017-0916-4 (PMC5706168; doi:10.1186/s13058-017-0916-4)
Supplement: Supplementary file 1 — Alleles analyzed by INFINITI®. (DOCX 14 kb) [file 13058_2017_916_MOESM1_ESM.docx]

**Additional file 1: Table S1. Alleles analyzed by INFINITI®**

| **Allele** | **Mutation** | **Effect** |
| --- | --- | --- |
| *1 | wildtype | Normal |
| *2 | 2850C>T | Normal |
| *2A | -1584C>G | Normal |
| *3 | 2549delA | None |
| *4 | 1846G>A | None |
| *5 | deletion | None |
| *6 | 1707delT | None |
| *7 | 2935A>C | None |
| *8 | 1758G>T | None |
| *9 | 2615_2617delAAG | Decreased |
| *10 | 100C>T | Decreased |
| *12 | 124G>A | None |
| *14 | 1758G>A | None |
| *17 | 1023C>T | Decreased |
| *29 | 1659G>A | Decreased |
| *41 | 2988G>A | Decreased |
| *XN | multiple | Increased |
