# Supplementary material for: Serum concentrations of active tamoxifen metabolites predict long-term survival in adjuvantly treated breast cancer patients
Source: Breast Cancer Res. 2017 Nov 28;19:125. doi: 10.1186/s13058-017-0916-4 (PMC5706168; doi:10.1186/s13058-017-0916-4)
Supplement: Supplementary file 2 — Suppliers and catalog numbers for tamoxifen metabolites. (DOCX 14 kb) [file 13058_2017_916_MOESM2_ESM.docx]

**Additional file 2: Table S2. Suppliers and catalog numbers for tamoxifen metabolites**

| **Analyte** | **Catalog number** | **Supplier** |
| --- | --- | --- |
| Tamoxifen | 85256 | Sigma Aldrich Norway (Oslo, Norway) |
| NDtam | D293900 | Toronto Research Chemicals Inc. ( Toronto, ON, Canada) |
| Z-Endoxifen | D292043 | Toronto Research Chemicals Inc. ( Toronto, ON, Canada) |
| Z-4’Endoxifen | D292041 | Toronto Research Chemicals Inc. ( Toronto, ON, Canada) |
| Z-4OHtam | sc-3542 | Santa Cruz Biotechnology (Dallas, TXU, USA) |
| 4'OHtam | D292041 | Toronto Research Chemicals Inc. ( Toronto, ON, Canada) |
| Tam-N-ox | T006095 | Toronto Research Chemicals Inc. ( Toronto, ON, Canada) |
| NNDDtam | D441200 | Toronto Research Chemicals Inc. ( Toronto, ON, Canada) |
| cis-β-OHtam | H954717 | Toronto Research Chemicals Inc. ( Toronto, ON, Canada) |
| z-α-OHtam | H954710 | Toronto Research Chemicals Inc. ( Toronto, ON, Canada) |
| Tamoxifen-d5 | T006077 | Toronto Research Chemicals Inc. ( Toronto, ON, Canada) |
| 4OHNDtam-d5 | H9547 | Toronto Research Chemicals Inc. ( Toronto, ON, Canada) |
| Z-4-OHtam-d5 | H954757 | Toronto Research Chemicals Inc. ( Toronto, ON, Canada) |
| ND-tam-d5 | D293902 | Toronto Research Chemicals Inc. ( Toronto, ON, Canada) |
