# Supplementary material for: Serum concentrations of active tamoxifen metabolites predict long-term survival in adjuvantly treated breast cancer patients
Source: Breast Cancer Res. 2017 Nov 28;19:125. doi: 10.1186/s13058-017-0916-4 (PMC5706168; doi:10.1186/s13058-017-0916-4)
Supplement: Supplementary file 3 — Imprecision and accuracy. (DOCX 16 kb) [file 13058_2017_916_MOESM3_ESM.docx]

**Additional file 3 Table S3. Imprecision and accuracy**

| **Analyte** | **Calibrator** | | **Concentration, nmol/L** | | **Within day CV, %** | | **Between day CV, %** | | **Accuracy, %** | |
| --- | --- | --- | --- | --- | --- | --- | --- | --- | --- | --- |
|  |  | |  | |  | |  | |  | |
| Tamoxifen | Low | | 99.83 | | 3.6 | | 2.9 | | 102 | |
|  | Medium | | 399.31 | | 2.6 | | 2.3 | | 101 | |
|  | High | | 798.63 | | 3.7 | | 2.4 | | 99 | |
| Tam-N-ox | Low | | 24.69 | | 5.7 | | 1.9 | | 98 | |
|  | Medium | | 98.75 | | 2.3 | | 3.2 | | 102 | |
|  | High | | 197.49 | | 3.3 | | 3.5 | | 103 | |
| Z-4’Endoxifen | Low | | 32.80 | | 7.9 | | 7.0 | | 100 | |
|  | Medium | | 131.20 | | 4.7 | | 6.5 | | 97 | |
|  | High | | 262.40 | | 8.5 | | 6.0 | | 101 | |
| Z-Endoxifen | Low | | 28.09 | | 10.5 | | 0.6 | | 106 | |
|  | Medium | | 112.34 | | 7.3 | | 2.6 | | 99 | |
|  | High | | 224.68 | | 5.7 | | 1.9 | | 100 | |
| NDtam | Low | | 200.10 | | 5.4 | | 1.4 | | 103 | |
|  | Medium | | 800.40 | | 5.0 | | 6.7 | | 100 | |
|  | High | | 1600.80 | | 5.6 | | 4.5 | | 95 | |
| NNDDtam | Low | | 49.67 | | 6.3 | | 8.7 | | 99 | |
|  | Medium | | 198.67 | | 4.5 | | 2.8 | | 99 | |
|  | High | | 397.33 | | 5.1 | | 0.8 | | 96 | |
| 4'OHtam | Low | | 2.40 | | 7.4 | | 6.4 | | 100 | |
|  | Medium | | 9.60 | | 4.5 | | 5.1 | | 105 | |
|  | High | | 19.2 | | 2.8 | | 3.8 | | 102 | |
| Z-4OHtam | Low | | 2.40 | | 7.4 | | 2.8 | | 97 | |
|  | Medium | | 9.60 | | 4.0 | | 2.2 | | 103 | |
|  | High | | 19.19 | | 3.9 | | 2.9 | | 98 | |
| z-α-OHtam | Low | | 2.00 | | 9.6 | | 12.4 | | 109 | |
|  | Medium | | 8.00 | | 8.2 | | 11.4 | | 101 | |
|  | High | | 16.00 | | 7.7 | | 14.1 | | 98 | |
| cis-β-OHtam | Low | | 2.00 | | 11.9 | | 3.3 | | 87 | |
|  | Medium | | 8.00 | | 8.3 | | 11.6 | | 94 | |
|  | High | | 16.00 | | 13.5 | | 5.0 | | 87 | |
|  | |  | |  | |  | |  | |  |
