# Supplementary material for: Serum concentrations of active tamoxifen metabolites predict long-term survival in adjuvantly treated breast cancer patients
Source: Breast Cancer Res. 2017 Nov 28;19:125. doi: 10.1186/s13058-017-0916-4 (PMC5706168; doi:10.1186/s13058-017-0916-4)
Supplement: Supplementary file 4 — Retention times, molecular weights, and compound-dependent instrument settings. (DOCX 16 kb) [file 13058_2017_916_MOESM4_ESM.docx]

**Additional file 4: Table S4. Retention times molecular weights and compound dependent instrument settings**

|  | | | | | | | | | | |
| --- | --- | --- | --- | --- | --- | --- | --- | --- | --- | --- |
|  | **Rt, min** | **Parent ion (Q1)** |  | **Quantifier ion (Q3)** | | |  | **Qualifier ion (Q3)** | | |
|  |  | **mass** |  | **mass** | **Cone, V** | **CE, V** |  | **mass** | **Cone, V** | **CE, V** |
| Tamoxifen | 4.31 | 372.23 |  | 71.97 | 22 | 22 |  | 128.96 | 22 | 24 |
| Tamoxifen-d5 | 4.30 | 377.10 |  | 72.00 | 22 | 22 |  | 129.00 | 22 | 24 |
| Tam-N-ox | 4.40 | 388.00 |  | 72.00 | 2 | 22 |  | 58.00 | 2 | 20 |
| Z-4’Endoxifen | 4.07 | 374.16 |  | 57.08 | 20 | 2 |  | 129.04 | 50 | 28 |
| Z-Endoxifen | 3.93 | 374.16 |  | 58.08 | 78 | 16 |  | 223.09 | 8 | 18 |
| N-4OHNDtam-d5 | 3.91 | 379.23 |  | 58.20 | 2 | 20 |  | 228.18 | 50 | 18 |
| NDtam | 4.29 | 358.00 |  | 58.00 | 40 | 18 |  | 129.00 | 40 | 22 |
| NDtam-d5 | 4.29 | 363.30 |  | 58.21 | 40 | 18 |  | 133.84 | 40 | 22 |
| NNDDtam | 4.29 | 344.00 |  | 207.00 | 56 | 14 |  | 129.00 | 56 | 18 |
| 4'OHtam | 4.08 | 388.19 |  | 72.01 | 50 | 22 |  | 129.02 | 50 | 26 |
| Z-4OHtam | 3.94 | 388.19 |  | 72.04 | 50 | 22 |  | 129.02 | 50 | 26 |
| z-α-OHtam | 3.71 | 388.22 |  | 370.21 | 6 | 14 |  | 72.20 | 6 | 22 |
| cis-β-OHtam | 3.82 | 388.22 |  | 72.20 | 2 | 22 |  | 205.11 | 2 | 22 |
| z-4-OHtam-d5 | 3.93 | 393.31 |  | 72.18 | 50 | 22 |  | 132.02 | 50 | 26 |
| Cone, cone voltage; CE, collision energy; mass; mono-isotopic mass, Rt, retention time. Dwell time is 11 ms and repeller is set at 1,38 kV for all compounds. | | | | | | | | | | |
