# Supplementary material for: Serum concentrations of active tamoxifen metabolites predict long-term survival in adjuvantly treated breast cancer patients
Source: Breast Cancer Res. 2017 Nov 28;19:125. doi: 10.1186/s13058-017-0916-4 (PMC5706168; doi:10.1186/s13058-017-0916-4)
Supplement: Supplementary file 5 — Linear dynamic range of the assay. (DOCX 14 kb) [file 13058_2017_916_MOESM5_ESM.docx]

**Additional file 5: Table S5. Linear dynamic range of the assay**

|  |  | **Regression parameters** | | |
| --- | --- | --- | --- | --- |
| **Analyte** | **Linear range, nM** | **Slope (SD)** | **Intercept (SD)** | **r^2^** |
| Tamoxifen | 12.4-798 | 0.059 | 0.175 | 0.998 |
| Tam-N-ox | 3.0-197 | 0.022 | 0.069 | 0.997 |
| Z-4'Endoxifen | 4.1-262 | 0.003 | 0.004 | 0.999 |
|  |  |  |  |  |
| Z-Endoxifen | 3.5-224 | 0.002 | -0.002 | 0.996 |
| NDtam | 25.0-1600 | 0.035 | 0.114 | 0.988 |
| NNDDtam | 6.2-397 | 0.003 | -0.01 | 0.997 |
| 4'OHtam | 0.3-19.2 | 0.016 | 0.006 | 0.997 |
| Z-4OHtam | 3.5-224 | 0.002 | -0.002 | 0.996 |
| z-α-OHtam | 0.2-16.0 | 0.007 | -0.002 | 0.994 |
| cis-β-OHtam | 0.2-16.0 | 0.016 | -0.001 | 0.996 |
|  |  |  |  |  |
