# Supplementary material for: Serum concentrations of active tamoxifen metabolites predict long-term survival in adjuvantly treated breast cancer patients
Source: Breast Cancer Res. 2017 Nov 28;19:125. doi: 10.1186/s13058-017-0916-4 (PMC5706168; doi:10.1186/s13058-017-0916-4)
Supplement: Supplementary file 6 — Additional methods. Description of methods to determine cut-off values. (DOCX 17 kb) [file 13058_2017_916_MOESM6_ESM.docx]

**Supplement**

***Methods***

*Determination of cut-off values for endoxifen, Z-4OHtam and anti-estrogenic activity scores*

Since survival endpoints are time dependent and the underlying clinical setting is truly multivariable, we considered a multivariable Cox model (highest Wald method) including all variables that were deemed useful when searching for an optimal cut-off value for Z-4OHtam and Z-endoxifen. This supervised method identified a plateau of several significant cut off values regarding Z-4OHtam and Z-endoxifen concentrations. To avoid selecting a cut off value that can lead to putative under-treatment of future patients, we considered the highest level of Z-4OHtam and Z-endoxifen (3.26 and 9.00 nM, respectively) as the most clinical relevant cut off values. In the same Cox model as above we confirmed a plateau of significant cut-off values for AAS. Based on the same view as for Z-4OHtam and Z-endoxifen above, we considered AAS=16.7 as the clinical most relevant cut off-value.
