# Supplementary material for: Serum concentrations of active tamoxifen metabolites predict long-term survival in adjuvantly treated breast cancer patients
Source: Breast Cancer Res. 2017 Nov 28;19:125. doi: 10.1186/s13058-017-0916-4 (PMC5706168; doi:10.1186/s13058-017-0916-4)
Supplement: Supplementary file 7 — CYP2D6 allele frequencies. (DOCX 13 kb) [file 13058_2017_916_MOESM7_ESM.docx]

**Additional file 7: Table S6. *CYP2D6* Allele frequencies**

| **Allele** | **Frequency** | **Frequency (%)** |
| --- | --- | --- |
| *1 | 79 | 35.9 |
| *2 | 51 | 23.2 |
| *3 | 3 | 1.36 |
| *4 | 45 | 20.5 |
| *5 | 11 | 5.00 |
| *6 | 2 | 0.91 |
| *9 | 8 | 3.64 |
| *10 | 3 | 1.36 |
| *41 | 18 | 8.18 |
