# Supplementary material for: Serum concentrations of active tamoxifen metabolites predict long-term survival in adjuvantly treated breast cancer patients
Source: Breast Cancer Res. 2017 Nov 28;19:125. doi: 10.1186/s13058-017-0916-4 (PMC5706168; doi:10.1186/s13058-017-0916-4)
Supplement: Supplementary file 8 — Concentrations of tamoxifen metabolites stratified by metabolizer group. (DOCX 14 kb) [file 13058_2017_916_MOESM8_ESM.docx]

**Additional file 8: Table S7. Concentrations of tamoxifen metabolites stratified by metabolizer group**

| **Analyte** | **PM** | **IM** | **EM** | **UM** |
| --- | --- | --- | --- | --- |
| Tamoxifen | 250.84 (161.06) | 298.22 (213.09) | 259.19 (231.79) | 232.54 (408.69) |
| TamNoX | 67.57 (67.45) | 109.67 (73.70) | 78.62 (120.17) | 64.77 (200.88) |
| Z-4'Endoxifen | 25.20 (36.54) | 29.70 (13.52) | 23.21 (17.79) | 21.30 (24.77) |
| Z-Endoxifen | 9.88 (6.23) | 18.98 (23.89) | 28.75 (30.00) | 37.82 (78.33) |
| NDtam | 744.80 (536.62) | 740.08 (466.04) | 501.64 (503.64) | 421.13 (605.02) |
| NNDDtam | 72.42 (98.17) | 79.33 (49.98) | 75.05 (87.22) | 58.44 (114.38) |
| Z-4’OHtam | 6.75 (8.50) | 7.27 (4.35) | 6.20 (4.20) | 6.12 (9.54) |
| Z-4OHtam | 4.00 (2.38) | 4.62 (2.91) | 5.38 (4.41) | 6.21 (9.83) |

Median (inter quantile range). Data are nmol/L.
