# Supplementary material for: Serum concentrations of active tamoxifen metabolites predict long-term survival in adjuvantly treated breast cancer patients
Source: Breast Cancer Res. 2017 Nov 28;19:125. doi: 10.1186/s13058-017-0916-4 (PMC5706168; doi:10.1186/s13058-017-0916-4)

**Additional file 9: Figure S1. Kaplan-Meier plot of breast cancer specific survival according to Anti-estrogenic activity score.** Patients grouped according to the Anti-estrogenic Activity Score (AAS) ≤/> 16.7 (p = 0.026; log-rank). AAS calculated by the following algorithm: 0.01 × [Tam] + 1 × [Z-endoxifen + Z-4-OH-Tam] + 0.1 × [Z′-endoxifen+ Z′-4-OH-Tam].


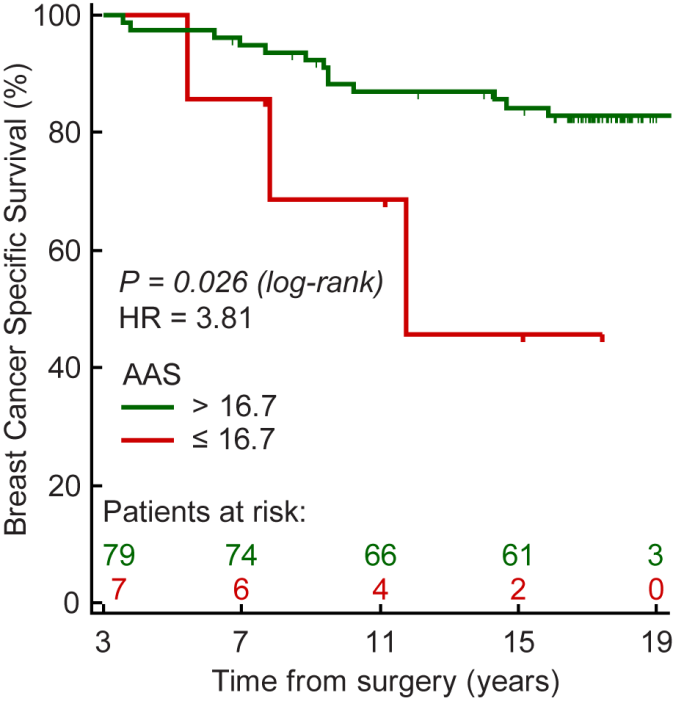

Supplement: Supplementary file 9 — Kaplan-Meier plot of breast cancer-specific survival according to Antiestrogenic Activity Score. (DOCX 109 kb) [file 13058_2017_916_MOESM9_ESM.docx]
