# Supplementary material for: Serum concentrations of active tamoxifen metabolites predict long-term survival in adjuvantly treated breast cancer patients
Source: Breast Cancer Res. 2017 Nov 28;19:125. doi: 10.1186/s13058-017-0916-4 (PMC5706168; doi:10.1186/s13058-017-0916-4)
Supplement: Supplementary file 10 — Distribution of patients with high and low Z-endoxifen (cut-off 9.0 nM) among patients with high and low Z-4OHtam (cut-off 3.26 nM). (DOCX 13 kb) [file 13058_2017_916_MOESM10_ESM.docx]

**Additional file 10: Table S8.** Distribution of patients with high and low Z-endoxifen (cutoff 9.0 nM) among patients with high and low Z-4OHtam (cutoff 3.26nM).

|  | **Endoxifen ≤ 9 nM** | **Endoxifen > 9 nM** | **Total** |
| --- | --- | --- | --- |
| **Z4OHtam ≤ 3.26 nM** | 5 (50.0 %) | 5 (50.0 %) | 10 (100 %) |
| **Z4OHtam > 3.26 nM** | 2 (2,6 %) | 74 (97,4 %) | 76 (100 %) |
| **Total** | 7 (8,1 %) | 79 (91,9 %) | 86 (100,0 %) |

Numbers represent count of patients in each group.
